# Longitudinal Trajectories of Platelet Indices Across Dengue Severity in Children: A Linear Mixed-Model Analysis

**DOI:** 10.7759/cureus.115217

**Published:** 2026-08-26

**Authors:** Balam Rishitha, Anjali Kale, Mohd Saeed Siddiqui, Pradnya M Joshi, Madhuri B Engade, Vinod Ingale, Murali Reddy, Akankshya Mahapatra, Apeksha Rajeardad

**Affiliations:** 1 Pediatrics, MGM Medical College and Hospital, MGM Institute of Health Sciences, Aurangabad, IND

**Keywords:** cpli, dengue fever (df), large platelet percentage, linear mixed model, platelet indices, thrombocytopenia

## Abstract

Background

Dengue has a high disease burden, and some affected children progress to severe dengue (SD), making early identification of deterioration a clinical priority. Automated platelet indices such as mean platelet volume (MPV), platelet distribution width (PDW), and plateletcrit (PCT) are generated at no additional cost by routine hematology analyzers. However, they are usually interpreted as static measurements and cannot distinguish peripheral platelet destruction from impaired bone marrow production. Linear mixed modeling (LMM) is well suited to correlated repeated measurements but has rarely been applied to platelet-index kinetics in pediatric dengue, particularly to distinguish dengue with warning signs (DWS) from SD. The objective of this study was to compare the longitudinal trajectories of eight automated platelet indices across dengue severity groups in hospitalized children using LMM and to assess whether platelet-index trajectories provide additional information beyond platelet count alone.

Methods

This prospective observational cohort included 130 children with dengue: 14 with dengue fever (DF), 80 with DWS, and 36 with SD, enrolled at a tertiary referral center. Platelet count, PCT, MPV, PDW, mean platelet component (MPC), mean platelet mass (MPM), large platelet percentage, and the composite platelet index (CPLI) were measured on study days 1, 3, 5, and 7. Study day 1 was the enrolment/admission day, which occurred within five days of fever onset. Separate LMMs were fitted for each index with fixed effects of severity group, illness day, age, sex, and a severity × day interaction, and a patient-level random intercept.

Results

Five indices were associated with dengue severity. Platelet count (day × severity interaction, p < 0.001), PCT (p < 0.001), large platelet percentage (p = 0.035), and CPLI (p < 0.001) showed significant severity-dependent trajectories. MPC was significantly associated with severity overall (p = 0.004), with a borderline day × severity interaction (p = 0.066). MPC showed progressive decline in SD from 26.1 g/dL (day 1) to 24.4 g/dL (day 5), signalling sustained platelet activation while remaining stable in DF. By day 3, CPLI was the only index with non-overlapping 95% confidence intervals between SD (1.46-3.74) and DWS (3.78-5.27). Large platelet percentage increased from 1.63% to 2.29% in DWS between study days 1 and 3 (absolute increase, 0.66 percentage points), compared with an increase from 1.18% to 1.28% in SD (absolute increase, 0.10 percentage points). This pattern is consistent with preserved compensatory thrombopoiesis in DWS and impaired compensation in SD. An exploratory day-1 profile of low platelet count, low CPLI, and a low large platelet percentage was observed in SD. MPV, PDW, and MPM did not discriminate by severity.

Conclusions

Platelet count, PCT, large platelet percentage, and CPLI showed severity-associated longitudinal trajectories in hospitalized children with dengue. The SD pattern is consistent with concurrent platelet consumption and impaired compensatory thrombopoiesis. These findings require multicenter validation, analyzer standardization, and formal prognostic evaluation before clinical application.

## Introduction

Dengue virus infection is a major global health burden, causing an estimated 390 million infections annually, predominantly in tropical and subtropical regions, including the Indian subcontinent and Southeast Asia [1]. Although most pediatric infections are self-limited, some progress to severe dengue (SD), characterized by plasma leakage, hemorrhage, or multi-organ dysfunction and associated with substantial morbidity, mortality, and health-system strain [2]. Early identification of children at risk is essential because timely supportive care reduces case fatality [3]. Risk stratification nevertheless continues to rely heavily on warning signs and serial platelet counts, which may lag the underlying pathophysiology and provide insufficient early discrimination [4].

Automated platelet indices, including mean platelet volume (MPV), platelet distribution width (PDW), and plateletcrit (PCT), are generated at no additional procedural cost by routine hematology analyzers and have attracted interest as prognostic markers in dengue [5,6]. These indices have proposed mechanistic relevance, though interpretation requires caution because MPV is influenced by multiple biological and analytical factors and is not itself a direct measure of thrombopoiesis. Elevated MPV may reflect compensatory thrombopoiesis and the release of larger, metabolically active platelets during peripheral immune-mediated destruction, whereas low MPV may indicate viral suppression of megakaryocyte production [5,7]. However, previous studies have yielded inconsistent findings, partly because platelet indices have generally been assessed at single time points. Values vary by illness day, and isolated measurements cannot distinguish peripheral destruction from impaired marrow production, which evolve differently during dengue [6,8]. This limits their clinical utility as severity markers [5,9]. Because accelerated platelet clearance and impaired megakaryocyte output may coexist [4,10], longitudinal assessment across the febrile and critical phases may better characterize the balance between platelet consumption and compensatory production [5,11].

Most previous dengue platelet-index studies have relied on cross-sectional comparisons or repeated-measures analyses conducted at fixed timepoints [6,9], which can inadequately address irregular sampling intervals and within-patient correlation when follow-up is incomplete or unevenly spaced [12,13]. Linear mixed models (LMMs) account for correlated observations, variable follow-up, and individual differences in temporal trajectories [13,14]. However, LMMs have rarely been applied to platelet-index kinetics in pediatric dengue, particularly to distinguish dengue with warning signs (DWS) from SD [9,11,15].

This study examined the longitudinal trajectories of platelet count, PCT, PDW, MPV, mean platelet mass (MPM), mean platelet component (MPC), large platelet percentage, and the composite platelet index (CPLI) across dengue severity groups in hospitalized children, assessed at protocol-definedstudy days 1, 3, 5, and 7 (distinct from illness day, i.e., day from fever onset, which varied at enrollment and is reported separately). Using LMMs, we sought to identify severity-associated kinetic differences, explore indirect evidence for the balance between platelet consumption and compensatory thrombopoiesis as inferred from platelet-index patterns, and determine whether platelet-index trajectories provide information beyond platelet count alone.

## Materials and methods

Study design and setting

This was a prospective observational study conducted at a tertiary pediatric referral center. Consecutive children admitted with suspected dengue infection were screened for eligibility and enrolled after written informed consent from parents or legal guardians. Ethical permission was obtained from the institutional ethics committee (MGM-ECRHS/2023/41; April 27, 2023).

Participants

Eligible patients were aged <18 years with laboratory-confirmed dengue, defined by non-structural protein 1 antigen (NS1 Ag) positivity or dengue immunoglobulin M (IgM) positivity, and were admitted within five days of fever onset. Immunoglobulin G (IgG) positivity alone was not considered sufficient to confirm acute dengue. Dengue severity was classified at discharge by the attending clinician according to the 2009 WHO criteria. DF was defined as dengue without warning signs, DWS as dengue with any warning sign, and SD as dengue with severe plasma leakage, severe bleeding, or severe organ dysfunction [5]. Because severity was determined at discharge, classification incorporated the patient's entire clinical course after admission; this study, therefore, evaluates platelet-index trajectories in relation to eventual (discharge) severity classification rather than testing whether early platelet indices prospectively predict subsequent SD. Routine clinical care was guided by standard clinical and laboratory assessment; study-specific platelet-index trajectories were not used to classify severity or direct management.

Inclusion criteria included patients with age <18 years, laboratory-confirmed dengue (NS1 antigen positivity or dengue IgM positivity), admission within five days of fever onset, and written informed consent obtained from a parent or legal guardian. Exclusion criteria included known pre-existing hematological disorders (e.g., congenital thrombocytopenia, leukemia), concurrent severe bacterial infection (positive sterile-site culture or sepsis requiring parenteral antibiotics), systemic immunosuppressive therapy within 30 days prior to admission, platelet transfusion prior to admission, and dengue IgG positivity alone (without NS1 or IgM confirmation).

Sample size

Sample size was determined a priori using G*Power version 3.1 (Heinrich-Heine-Universität Düsseldorf, Düsseldorf, Germany) for a repeated-measures within-between interaction analysis of variance (ANOVA) as an approximation of the LMM approach. Based on a partial η² of 0.052 from published pediatric dengue platelet-index data [9,16], corresponding to a Cohen’s f of 0.234, the calculation assumed three severity groups, four measurements, a within-subject correlation of 0.35, and a non-sphericity correction of ε = 0.5. A minimum sample size of 105 was required for 95% power at a two-sided α of 0.05. Allowing for 15% dropout or incomplete follow-up, the target sample was 130 participants. This calculation approximates the primary LMM: repeated-measures ANOVA assumes a compound-symmetric covariance structure and does not include covariates, whereas the LMMs used a random-intercept covariance structure and adjusted for age and sex, so achieved power for the eight individual models may differ from this a priori estimate.

Measurements

Peripheral venous blood was collected in ethylene diamine tetraacetic acid tubes on study days 1, 3, 5, and 7. Study day 1 was the enrollment or admission day, occurring within five days of fever onset; subsequent samples were targeted approximately 48 hours apart. The corresponding illness day from fever onset was recorded for each sample. Study day, rather than illness day, was used as the time metric in all LMMs and figures, because it reflected the protocol-defined sampling schedule common to all participants.

Samples were processed within two hours using a Siemens ADVIA 2120 analyzer (Siemens Healthineers, Erlangen, Germany) according to the manufacturer’s instructions. Samples with analyzer flags indicating platelet clumping or unreliable indices were repeated or excluded, and exclusions were documented. Internal quality control, maintenance, and calibration followed the manufacturer’s schedule. Laboratory personnel were blinded to clinical severity and outcomes.

Eight indices were recorded: platelet count (×10³/μL), PCT (%), MPV (fL), PDW (%), MPC (g/dL), MPM (pg), large platelet percentage (%), and CPLI, calculated as platelet count expressed in 10⁵/µL multiplied by MPV in fL. Hemoglobin, hematocrit, and white cell count were measured concurrently. Intensive care unit admission, hospital stay, and mortality were extracted by a team member blinded to platelet-index values.

Statistical analysis

Continuous variables are presented as mean ± standard deviation or median with interquartile range, and categorical variables as number and percentage. Baseline differences among DF, DWS, and SD were assessed using ANOVA or the Kruskal-Wallis test for continuous variables and the chi-squared or Fisher’s exact test for categorical variables.

Separate LMMs were fitted for each platelet index. Fixed effects included severity group, with DF as the reference, study day, age, sex, and the severity × day interaction. Study day was modeled as a continuous variable in the primary analysis, and a patient-level random intercept accounted for within-subject correlation. Random slopes for study day were considered but were not retained because they did not improve model fit or failed to converge. Degrees of freedom were estimated using the Satterthwaite approximation. Although study day was modeled as a continuous fixed effect, the day × severity interaction and severity main-effect F tests reported in the results are the omnibus Type III tests from the GAMLj mixed-model ANOVA output; estimated marginal means (EMMs) at study days 1, 3, 5, and 7 represent model-predicted values at those specific days rather than a categorical day effect. Model performance was summarized using marginal and conditional R² values.

Model assumptions were evaluated by inspecting residuals and random effects, with no major violations of normality or homoscedasticity identified. Post-hoc EMMs with 95% confidence intervals were calculated for study days 1, 3, 5, and 7, adjusted to the cohort mean age and sex. Pairwise between-group contrasts at individual time points were interpreted descriptively; when formally tested, p-values were adjusted for multiple comparisons. Statistical significance was set at a two-sided p < 0.05. No family-wise correction was applied because the eight indices were prespecified tests addressing a single overarching research question rather than independent post hoc comparisons; given the number of indices tested, however, the three non-significant indices (MPV, PDW, MPM) and the borderline MPC interaction are interpreted as exploratory rather than confirmatory. Analyses were performed using jamovi version 2.4.1 (The jamovi project, Sydney, New South Wales, Australia) with the GAMLj module.

## Results

Patient characteristics

One hundred and thirty patients were enrolled: 14 (10.8%) DF, 80 (61.5%) DWS, and 36 (27.7%) SD. SD patients were significantly younger (5.4 ± 5.5 vs. 9.6 ± 5.2 years in DWS; p = 0.001). ICU admission was near-universal in SD (34, 94.4%) vs. in DWS (2, 2.5%), and in-hospital mortality occurred exclusively in SD (4/36, 11.1%; p = 0.005). Admission platelet count, PCT, and CPLI were lowest in SD, though differences were not statistically significant at day 1 (all p > 0.05; Table 1). Platelet transfusion was administered to 24 of 130 patients (18.5%). No patient in the dengue fever (DF) group required transfusion, whereas six of 80 patients (7.5%) with DWS received a single transfusion, and 18 of 36 patients (50%) with SD received transfusions (11 received one unit and seven received two units).

**Table 1:** Baseline characteristics and admission platelet indices by dengue severity Continuous variables are mean ± standard deviation, compared by analysis of variance (ANOVA) with the corresponding F statistic value; categorical variables are n (%), compared by the chi-squared or Fisher's exact test (with the corresponding chi-squared statistic value); degrees of freedom (df) are shown in parentheses after χ². DF: dengue fever; DWS: dengue with warning signs; SD: severe dengue (as a group label: not to be confused with "SD" denoting standard deviation in ± SD values); ICU: intensive care unit; PCT: plateletcrit; MPV: mean platelet volume; PDW: platelet distribution width; MPC: mean platelet component; MPM: mean platelet mass; CPLI: composite platelet index (platelet count × MPV)

Variable	DF (n = 14)	DWS (n = 80)	SD (n = 36)	Statistic value	p-value
Age, y (mean ± SD)	8.0 ± 6.3	9.6 ± 5.2	5.4 ± 5.5	7.24	0.001
Female sex, n (%)	6 (42.9)	36 (45.0)	21 (58.3)	χ²(2) = 3.01	0.557
Platelet count ×10³/μL	65.3 ± 29.2	56.8 ± 33.0	45.9 ± 30.2	2.31	0.104
PCT % (mean ± SD)	0.05 ± 0.03	0.04 ± 0.02	0.03 ± 0.02	2.64	0.075
MPV fL (mean ± SD)	7.4 ± 1.2	7.2 ± 1.2	7.2 ± 1.3	0.16	0.848
PDW % (mean ± SD)	51.9 ± 18.5	55.9 ± 19.4	52.3 ± 21.5	0.52	0.594
MPC g/dL (mean ± SD)	26.5 ± 2.1	26.7 ± 2.0	25.8 ± 1.9	2.38	0.097
MPM pg (mean ± S D)	1.9 ± 0.3	1.8 ± 0.2	1.7 ± 0.3	1.28	0.282
Large platelet % (mean ± SD)	2.1 ± 3.4	1.6 ± 2.1	1.2 ± 1.4	0.89	0.413
CPLI (mean ± SD)	5.0 ± 2.7	4.1 ± 2.5	3.3 ± 2.2	2.56	0.081
ICU admission, n (%)	0 (0.0)	2 (2.5)	34 (94.4)	χ²(2) = 110.83	<0.001
In-hospital death, n (%)	0 (0.0)	0 (0.0)	4 (11.1)	χ²(2) = 10.78	0.005

Discriminating indices

**Figure 1:**
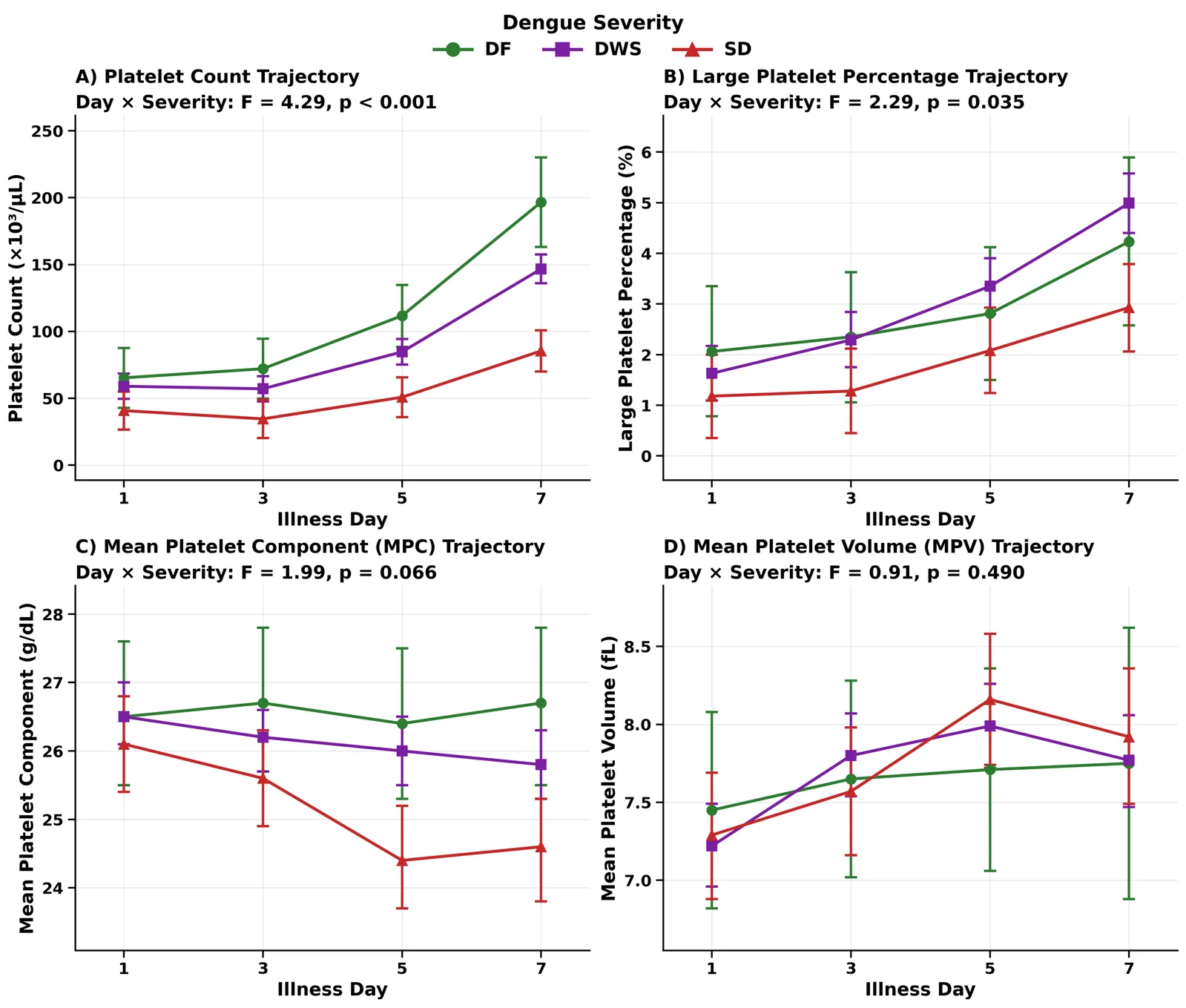
Longitudinal trajectories of platelet count, large platelet %, MPC and MPV Estimated marginal mean trajectories (error bars: 95% CI) across illness days 1, 3, 5, and 7 for DF: dengue fever; DWS: dengue with warning signs; and SD: severe dengue (A) Platelet count (×10³/μL); (B) large platelet percentage; (C) mean platelet component (MPC, g/dL); (D) mean platelet volume (MPV, fL). F and p values in each panel denote the illness day × severity interaction from the corresponding linear mixed model.

All eight LMMs converged successfully using 478 of a theoretical maximum of 520 observations. Missing laboratory data were absent at day 1 across all severity groups, remained minimal at day 3 (0 in DF and DWS; 1/36 in SD), and increased thereafter, reaching 1/14 (7%) at day 5 and 8/14 (57%) at day 7 in DF, 3/80 (4%) at day 5 and 19-21/80 (24-26%) at day 7 in DWS, and 3/36 (8%) at day 5 and 6-7/36 (17-19%) at day 7 in SD, primarily reflecting earlier hospital discharge in milder cases. Significant day × severity interactions were observed for platelet count (F = 4.29, p < 0.001), PCT (F = 3.87, p < 0.001), large platelet percentage (F = 2.29, p = 0.035), and CPLI (F = 3.81, p < 0.001). MPC showed a significant overall severity effect (F = 5.65, p = 0.004), with a borderline day × severity interaction (F = 1.99, p = 0.066). MPV, PDW, and MPM did not show statistically significant severity-associated trajectories (all interaction p > 0.05; Table 2). Conditional R² for these three exploratory indices ranged from 0.495 to 0.586, compared with 0.516-0.677 for the five severity-associated indices. Longitudinal trajectories of platelet count, large platelet percentage, MPC, and MPV are shown in Figure 1.

**Table 2:** Linear mixed model (LMM) fit statistics for all eight platelet indices PCT: plateletcrit; MPV: mean platelet volume; PDW: platelet distribution width; MPC: mean platelet component; MPM: mean platelet mass; CPLI: composite platelet index (platelet count × MPV). Marg. R²: marginal R-squared (variance explained by fixed effects alone); Cond. R²: conditional R-squared (fixed + random effects). Day × Severity F/p value: the illness day-by-severity interaction term; Severity p value: the overall main effect of the severity group. MPC was significant as a severity main effect (p = 0.004) despite a borderline day × severity interaction (p = 0.066).

Index	Marg. R²	Cond. R²	Day × severity F statistic	Day × severity p-value	Severity F statistic	Severity p-value
Platelet count	0.402	0.52	4.29	<0.001	25.45	<0.001
PCT	0.386	0.536	3.87	<0.001	20.9	<0.001
Large platelet %	0.18	0.677	2.29	0.035	3.75	0.026
CPLI	0.394	0.551	3.81	<0.001	20.91	<0.001
MPC	0.186	0.516	1.99	0.066	5.65	0.004
MPV	0.064	0.495	0.91	0.49	0.05	0.953
PDW	0.081	0.536	0.29	0.941	0.82	0.444
MPM	0.098	0.586	0.7	0.648	1.63	0.2

Severity-stratified trajectories and exploratory day 1 patterns

EMMs for the five severity-associated indices are presented in Table 3. At study day 1, EMM platelet count and PCT were approximately 30% lower in SD than in DWS. Between study days 1 and 3, the EMM platelet count decreased from 40.8 to 34.6 ×10³/µL in SD (absolute change, -6.2 × 10³/µL) and from 59.0 to 57.1 × 10³/µL in DWS (absolute change, -1.9 × 10³/µL). PCT was essentially unchanged in SD after rounding (0.029% to 0.029%) and increased from 0.043% to 0.046% in DWS. By day 5, platelet count remained approximately 40% lower in SD than DWS (50.8 vs. 84.7 × 10³/μL), a gap that persisted to day 7, when SD values remained suppressed relative to DWS (platelet count 85.4 × 10³/μL vs 146.7 × 10³/μL).

**Table 3:** Estimated marginal means (95% CI) for discriminating platelet indices at each illness day by dengue severity Values are estimated marginal means (95% CI) from the linear mixed models, adjusted to cohort mean age and sex. DF: dengue fever; DWS: dengue with warning signs; SD: severe dengue. PCT: plateletcrit; MPC: mean platelet component; CPLI: composite platelet index (platelet count × MPV); Large PLT%: large platelet percentage

Day	Group	n	Platelet count (×10³/µL)	PCT (%)	Large PLT% (%)	CPLI	MPC (g/dL)
1	DF	14	65.3 (42.9-87.6)	0.049 (0.03-0.07)	2.06 (0.78-3.35)	4.96 (3.19-6.73)	26.5 (25.5-27.6)
1	DWS	80	59.0 (49.5-68.4)	0.043 (0.04-0.05)	1.63 (1.09-2.17)	4.27 (3.52-5.01)	26.5 (26.1-27.0)
1	SD	36	40.8 (26.6-55.0)	0.029 (0.02-0.04)	1.18 (0.35-2.00)	2.93 (1.81-4.06)	26.1 (25.4-26.8)
3	DF	14	72.1 (49.7-94.5)	0.056 (0.04-0.07)	2.35 (1.06-3.63)	5.51 (3.74-7.27)	26.7 (25.7-27.8)
3	DWS	80	57.1 (47.7-66.5)	0.046 (0.04-0.05)	2.29 (1.75-2.84)	4.52 (3.78-5.27)	26.2 (25.7-26.6)
3	SD	35	34.6 (20.2-49.0)	0.029 (0.02-0.04)	1.28 (0.45-2.12)	2.60 (1.46-3.74)†	25.6 (24.9-26.3)
5	DF	13	111.7 (88.5-134.8)	0.084 (0.06-0.10)	2.81 (1.50-4.12)	8.29 (6.47-10.12)	26.4 (25.3-27.5)
5	DWS	77	84.7 (75.1-94.3)	0.069 (0.06-0.08)	3.35 (2.80-3.90)	6.80 (6.05-7.56)	26.0 (25.5-26.5)
5	SD	33	50.8 (36.0-65.6)	0.042 (0.03-0.05)	2.08 (1.24-2.93)	4.23 (3.06-5.40)	24.4 (23.7-25.2)
7	DF	6	196.6 (163.2-229.9)	0.152 (0.12-0.18)	4.23 (2.58-5.89)	15.06 (12.47-17.66)	26.7 (25.5-27.8)
7	DWS	61	146.7 (136.0-157.4)	0.113 (0.10-0.12)	4.99 (4.40-5.58)	11.27 (10.43-12.11)	25.8 (25.3-26.3)
7	SD	30	85.4 (69.9-100.8)	0.067 (0.05-0.08)	2.93 (2.06-3.79)	6.69 (5.46-7.92)	24.6 (23.8-25.3)

The most striking divergence was in thrombopoietic compensation. Large platelet percentage increased from 1.63% to 2.29% in DWS between study days 1 and 3 (absolute increase, 0.66 percentage points), eventually exceeding DF by day 7, whereas SD increased from 1.18% to 1.28% (absolute increase, 0.10 percentage points) despite worse thrombocytopenia. This pattern is consistent with relatively preserved compensatory thrombopoiesis in DWS and impaired compensation in SD. CPLI decreased from 2.93 to 2.60 in SD and increased from 4.27 to 4.52 in DWS over the same interval. At study day 3, the EMM CPLI was 2.60 (95% CI, 1.46-3.74) in SD and 4.52 (95% CI, 3.78-5.27) in DWS. The confidence intervals did not overlap, suggesting separation between these groups at this time point; however, this non-overlap should be interpreted descriptively rather than as a formal test of between-group significance. The EMM MPC in SD decreased from 26.1 g/dL on study day 1 to 24.4g/dL on study day 5, whereas estimates remained relatively stable in DF. Because the day × severity interaction for MPC was borderline, this pattern should be interpreted as a severity-associated trend rather than definitive evidence of a divergent trajectory.

As an exploratory observation, a day 1 pattern of platelet count below 45,000/µL, CPLI below 3.0, and large platelet percentage below 1.3% was observed among patients classified as SD. These thresholds were derived from the observed cohort and were not validated; they should be considered hypothesis-generating rather than diagnostic criteria.

## Discussion

This study shows that longitudinal platelet morphology and mass indices reveal severity-dependent patterns in pediatric dengue that admission platelet count alone may not capture. Platelet count, PCT, large platelet percentage, and CPLI demonstrated significant severity-associated trajectories, while MPC differed overall by severity. Collectively, the findings support a model in which DWS is characterized primarily by platelet consumption with relatively preserved compensatory thrombopoiesis, whereas SD may involve both consumption and impaired compensatory production. Because platelet indices are indirect, correlative measures of platelet turnover rather than direct assays of marrow activity or peripheral destruction, they cannot, by themselves, establish impaired thrombopoiesis or platelet destruction.

The principal divergence between SD and DWS occurred early, between study days 1 and 3. Platelet count continued to decline in SD but changed little in DWS, with PCT following a similar pattern. This agrees with evidence that PCT reflects total platelet mass and may outperform platelet count alone as a severity marker [11,15,17,18]. The present analysis extends those observations by demonstrating a continuous severity-by-time interaction rather than relying on a single measurement or threshold.

MPV, PDW, and MPM did not discriminate among severity groups, consistent with the mixed findings of previous studies. Some have reported increasing MPV or PDW with greater severity [8,19], whereas others found little prognostic value [20,21]. By evaluating complete trajectories, our LMM approach reduces the limitations of single-time-point comparisons. Despite lower platelet counts, SD was not accompanied by a compensatory rise in MPV. This may indicate impaired marrow output rather than predominantly peripheral destruction, although direct measures of marrow activity were unavailable [5,22]. PDW showed a similarly non-discriminating pattern, and MPM remained stable across groups, adding longitudinal evidence for an index rarely studied in dengue.

MPC declined progressively in SD, from 26.1 g/dL on study day 1 to 24.4 g/dL on day 5, while remaining comparatively stable in DF. To our knowledge, this is the first longitudinal description of MPC across WHO 2009 dengue severity categories. However, because the day × severity interaction was borderline, MPC should be interpreted as a severity-associated marker rather than definitive evidence of divergent trajectories. Previous dengue studies have not evaluated MPC directly. In vitro work by Macey et al. showed that thrombin-induced platelet activation produces a dose-dependent decline in MPC that correlates strongly with CD62P expression, supporting MPC as a marker of platelet activation and degranulation [23]. Our findings suggest a corresponding clinical severity gradient. In SD, declining MPC accompanied by limited increases in large platelet percentage and CPLI may indicate concurrent platelet activation or consumption and inadequate compensatory production, consistent with immune-mediated destruction and viral suppression of megakaryocytes [24,25].

Large platelet percentage showed a particularly informative divergence. Between study days 1 and 3, it increased from 1.63% to 2.29% in DWS but only from 1.18% to 1.28% in SD, despite more marked thrombocytopenia in the latter. This pattern suggests reduced compensatory megakaryopoiesis in SD. It is consistent with platelet large cell ratio findings reported by Guo et al., who observed an inverse correlation between this measure and platelet count, and by Asha et al., who reported higher values in transfused than non-transfused patients with dengue [6,11]. Our results suggest that the magnitude and timing of the compensatory response, rather than its presence alone, may distinguish severity - an effect that cross-sectional analyses may miss.

CPLI further emphasized this divergence. On study day 3, the confidence intervals for SD (1.46-3.74) and DWS (3.78-5.27) did not overlap. Although this suggests separation at that time point, confidence-interval non-overlap is not equivalent to a formal test of between-group significance. Krishnamurthy et al. introduced CPLI as a composite bleeding-risk index, reporting a median of 5.9 in dengue compared with 27.2 in controls, while Doreswamy et al. reported lower values in pediatric hemorrhagic shock [24,25]. The present study extends this work by evaluating CPLI across WHO 2009 severity groups and modeling its longitudinal trajectory with patient-level random effects. The high conditional R² for large platelet percentage also indicates substantial individual variation beyond severity group, potentially related to factors such as viral load and immune phenotype, consistent with associations between viral replication and platelet kinetics reported by Guo et al. [11].

Taken together, the findings suggest that DWS and SD may represent different mechanistic patterns of dengue-associated thrombocytopenia. DWS appears relatively consumption-dominant: immune-mediated destruction, endothelial sequestration, and complement activation reduce platelet numbers, but bone marrow compensation remains active. In SD, direct or indirect suppression of megakaryopoiesis may restrict the compensatory response. Thus, two patients with similar platelet counts - one with rising large platelet percentage and CPLI and another with flat or declining values - could have different trajectories and monitoring requirements.

The exploratory day 1 combination of platelet count below 45,000/µL, CPLI below 3.0, and large platelet percentage below 1.3% may identify children with severe thrombocytopenia, low composite platelet production, and limited large-platelet release. Similarly, falling or non-rising CPLI, failure of PCT to increase, and a limited rise in large platelet percentage between study days 1 and 3 may signal deterioration. However, these thresholds were derived from the study cohort and should not be used for diagnosis or clinical decision-making without prospective external validation.

Study strengths include the prospective longitudinal design, repeated sampling across the critical phase, use of LMMs, and simultaneous assessment of multiple platelet indices. Several limitations should be acknowledged. First, the single-center design and small DF subgroup limit generalizability and the precision of DF estimates. Second, severity was assigned clinically at discharge; although study indices were not used for classification, misclassification remains possible. Third, missing later samples may have been informative, particularly for children who died or were discharged early. Observation counts by study day and severity group are reported in the Results. Fourth, variation in illness day at admission may have introduced residual confounding by disease stage. Fifth, platelet indices differ across analyzer platforms and may be less reliable at very low platelet counts, limiting the generalizability of the proposed thresholds. Sixth, platelet transfusions after admission may have affected subsequent measurements, especially in SD. Seventh, thrombopoietin, immature platelet fraction, and bone marrow markers were not measured; therefore, impaired marrow compensation was inferred rather than directly demonstrated.

Future multicenter studies should standardize analyzer-specific measurements, document and adjust for post-admission platelet transfusions, and externally validate prespecified thresholds. Formal prognostic analyses are also needed to determine whether CPLI, large platelet percentage, and their trajectories improve existing dengue severity models.

## Conclusions

Longitudinal trajectories of platelet indices provide clinically relevant information about the balance between platelet consumption and compensatory thrombopoiesis that platelet count alone may not capture. Platelet count, PCT, large platelet percentage, and CPLI showed severity-associated longitudinal trajectories, while MPC differed overall by severity. The SD pattern is consistent with both platelet consumption and impaired compensatory thrombopoiesis. These findings are indirect and hypothesis-generating and require multicenter validation, analyzer standardization, and formal prognostic evaluation before clinical use.
